# Abdominal subcutaneous fat quantification in obese patients from limited field-of-view MRI data

**DOI:** 10.1038/s41598-020-75985-8

**Published:** 2020-11-04

**Authors:** Sophia Michel, Nicolas Linder, Tobias Eggebrecht, Alexander Schaudinn, Matthias Blüher, Arne Dietrich, Timm Denecke, Harald Busse

**Affiliations:** 1 Integrated Research and Treatment Center (IFB) Adiposity Diseases, Leipzig University Medical Center, Leipzig, Germany; 2 Department of Diagnostic and Interventional Radiology, Leipzig University Hospital, Liebigstrasse 20, 04103 Leipzig, Germany; 3 Department of Internal Medicine, Neurology and Dermatology, Division of Endocrinology and Nephrology, Leipzig University Hospital, Leipzig, Germany; 4 Department of Visceral, Transplantation, Thoracic and Vascular Surgery, Division of Bariatric Surgery, Leipzig University Hospital, Leipzig, Germany

**Keywords:** Biomarkers, Predictive markers, Metabolic disorders, Body mass index, Weight management

## Abstract

Different types of adipose tissue can be accurately localized and quantified by tomographic imaging techniques (MRI or CT). One common shortcoming for the abdominal subcutaneous adipose tissue (ASAT) of obese subjects is the technically restricted imaging field of view (FOV). This work derives equations for the conversion between six surrogate measures and fully segmented ASAT volume and discusses the predictive power of these image-based quantities. Clinical (gender, age, anthropometry) and MRI data (1.5 T, two-point Dixon sequence) of 193 overweight and obese patients (116 female, 77 male) from a single research center for obesity were analyzed retrospectively. Six surrogate measures of fully segmented ASAT volume (*V*_ASAT_) were considered: two simple ASAT lengths, two partial areas (*A*_p-FH_, *A*_p-ASIS_) and two partial volumes (*V*_p-FH_, *V*_p-ASIS_) limited by either the femoral heads (FH) or the anterior superior iliac spine (ASIS). Least-squares regression between each measure and *V*_ASAT_ provided slope and intercept for the computation of estimated ASAT volumes (*V*^~^_ASAT_). Goodness of fit was evaluated by coefficient of determination (*R*^2^) and standard deviation of percent differences (*s*_d%_) between *V*^~^_ASAT_ and *V*_ASAT_. Best agreement was observed for partial volume *V*_p-FH_ (*s*_d%_ = 14.4% and *R*^2^ = 0.78), followed by *V*_p-ASIS_ (*s*_d%_ = 18.1% and *R*^2^ = 0.69) and AWF_ASIS_ (*s*_d%_ = 23.9% and *R*^2^ = 0.54), with minor gender differences only. Other estimates from simple lengths and partial areas were moderate only (*s*_d%_ > 23.0% and *R*^2^ < 0.50). Gender differences in *R*^2^ generally ranged between 0.02 (*d*_ven_) and 0.29 (*A*_p-FH_). The common FOV restriction for MRI volumetry of ASAT in obese subjects can best be overcome by estimating *V*_ASAT_ from *V*_p-FH_ using the equation derived here. The very simple AWF_ASIS_ can be used with reservation.

## Introduction

Obesity is one of the major healthcare problems of the twenty-first century with a steadily increasing prevalence over the last decades^1^. In the United States, for example, data from the years 2013 to 2016 have shown that more than two-third of the adults were either obese (38.9%, BMI: 30–40 kg/m^2^) or severely obese (7.6%, BMI: > 40 kg/m^2^)^2^. Overweight and obesity are closely associated with an increased overall mortality and morbidity often caused by metabolic or cardiovascular diseases. Direct and indirect treatment costs generate an enormous socio-economic burden on society^3–5^.

Abdominal subcutaneous (ASAT) and visceral adipose tissue (VAT) contribute differently to metabolic homeostasis. Fat amounts and their distribution are important risk factors in the pathogenesis of cardiometabolic diseases^6^. VAT is an extremely dyslipidemic and atherogenic fat depot due to its endocrine activity^7^. In contrast, accumulation of ASAT is an independent predictor of lower cardiometabolic mortality^8^. Metabolic complications may arise when ASAT fails to expand and store fat leading to ectopic fat deposition with subsequent lipotoxicity and insulin resistance^9,10^. In view of limited health resources, there is a considerable interest in identifying robust predictors for cardiometabolic risk stratification and monitoring of clinical outcome in obese patients. The amount and distribution of ASAT and VAT are currently among the most promising parameters for that purpose. A weight-loss study by Mayo-Smith et al. has found that the relative volume reduction in ASAT was larger than in VAT, suggesting that ASAT might better reflect the nutritional status^11^.

Imaging techniques enable a non-invasive identification of individual fat compartments. MRI provides an excellent soft-tissue contrast and is characterized by the absence of ionizing radiation and should therefore be preferred for younger patients or longitudinal studies with repeated assessments^12–14^. Since the beginning of MRI-based fat quantification in the mid-1980s, methods have been validated post mortem against sections of human and animal cadavers^15–18^. A number of studies have argued that MRI should be preferred over CT in terms of safety^19–22^.

For subjects with a higher degree of obesity, it is common that the field of view (CT or MRI) is technically not large enough to image the whole cross section. With most patients being examined in supine position, this will typically exclude ventral or lateral subcutaneous adipose tissue and prevent exact quantification. This shortcoming, although referred to in the literature, has not been resolved so far. In our study, for example, the field of view was not large enough for about 82% of our patients.

The objective of this study was therefore to determine the power of various surrogate measures (distances, fat areas and partial fat volumes) for the prediction of total ASAT volume in overweight or obese adults with missing ('out-of-view') fat data. Gender-specific (linear) equations and measures of agreement are provided to identify more (or less) suitable approaches of ASAT volume estimation.

## Material and methods

### Study design and data

This retrospective IRB-approved study was performed at a single center–the Integrated Research and Treatment Center for Adiposity Diseases, University Medicine Leipzig, Leipzig, Germany. Data collection, analysis and publication were approved by the Institutional Review Board (IRB) of the Leipzig University Faculty of Medicine, Leipzig, Germany (reference numbers 283/11-ff, 284/10-ff, 363/10-ff, 363/11-ff) and informed consent was obtained from all subjects. All methods were carried out in accordance with relevant guidelines and regulations (Declaration of Helsinki).

**Figure 1:**
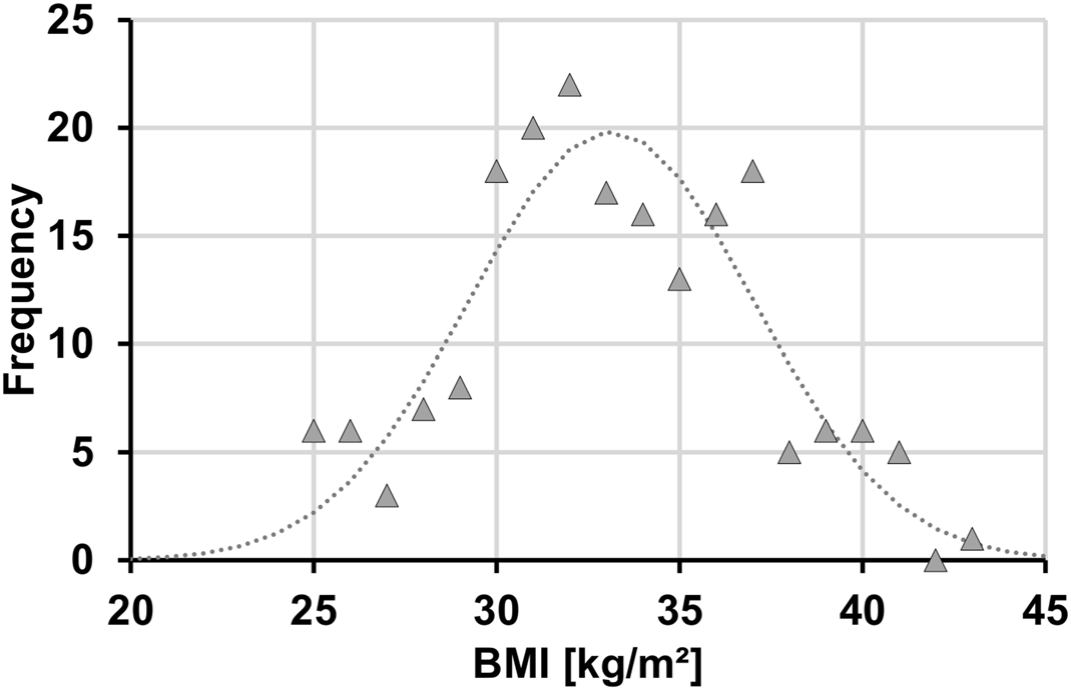
Histogram of BMI distribution for all 193 patients (range 24.8–41.2 kg/m^2^). Triangles at integer BMI values reflect number of patients (frequency) with actual BMI ± 0.5 kg/m^2^ around that value. Dotted curve indicates Gaussian fit to BMI data (32.1 ± 3.7 kg/m^2^).

A total of 193 patients (116 females and 77 males) were selected, primarily with respect to age (at least 18 years) and BMI (> 24.5 kg/m^2^). Patients with visibly missing parts of the abdominal subcutaneous tissue on any of the image slices were excluded. Figure 1 shows a histogram of the BMI distribution for all 193 patients along with a Gaussian fit to the data. Further details of the study population are given in Supplementary Table S1**.**

### MR imaging and analysis

**Figure 2:**
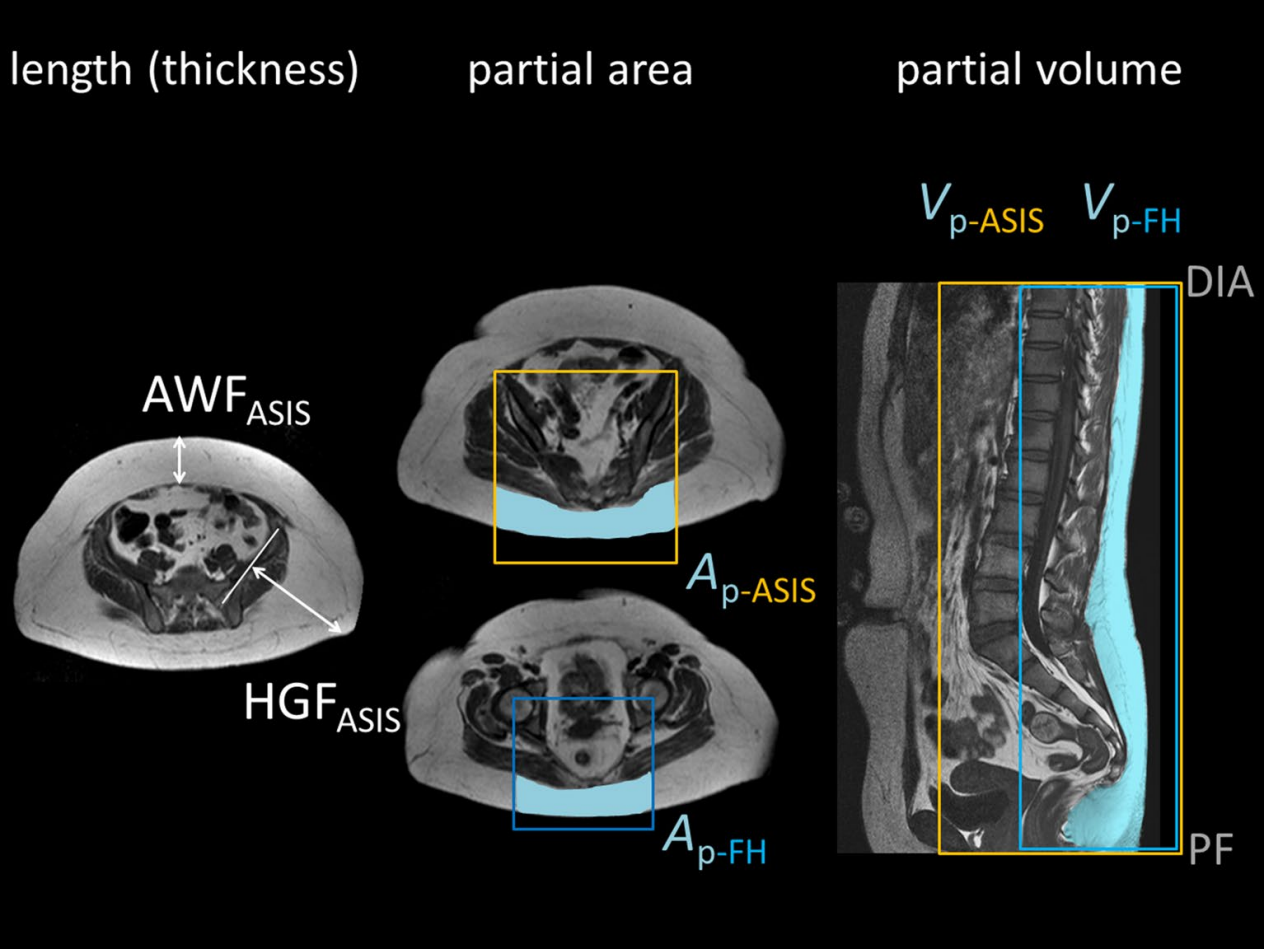
Overview of surrogate parameters. left: length (thickness) of abdominal wall fat (AWF_ASIS_) and hip girdle fat (HGF_ASIS_) at the level of the anterior superior iliac spine (ASIS). middle: partial ASAT area within rectangles bound by the femoral heads or ASIS (*A*_p -FH_, *A*_p-SIAS_). right: partial ASAT volume (*V*_p-FH_, *V*_p-ASIS_) is the sum over all local ASAT volumes (partial ASAT areas *A*_p_ × interslice spacing of 10.5 mm) between diaphragm and pelvic floor.

The patient was examined in supine position using a standard 1.5 T system (Achieva XR, Philips Healthcare, Best, Netherlands) and the integrated whole-body coil for signal reception. Fat-sensitive imaging was based on an axial two-point Dixon sequence with two stacks of 25 10-mm-thick slices (0.5 mm interslice gap) covering the whole abdominal cavity. The main imaging parameters are listed in Supplementary Table S2. Image analysis was performed with a custom made software^23–25^ that was developed using a predefined framework for radiological image analysis^26^. Surrogate measures of full ASAT volume were thickness of the abdominal wall fat or hip girdle fat (AWF and HGF), partial ASAT areas (*A*_p-FH_ and *A*_p-ASIS_) and partial volumes (*V*_p-FH_ and *V*_p-ASIS_). Nomenclature and definitions are given in Fig. 2 and Table 1**.**

**Table 1:** Nomenclature of surrogate parameters.

Quantity	Symbol	Definition
Reference	*V*_ASAT_	ASAT volume measured from segmented ASAT areas of *all slices* between diaphragm and pelvic floor (typically 40–50 slices with effective spacing of 10.5 mm)
Length (thickness)	*V*^~^_ASAT_(AWF_ASIS_)	ASAT volume estimated by length (thickness) of abdominal wall fat (AWF_ASIS_) or hip girdle fat (HGF_ASIS_) on *single slice* (at the level of the anterior superior iliac spine, ASIS)
	*V*^~^_ASAT_(HGF_ASIS_)	
Partial area	*V*^~^_ASAT_(*A*_p-ASIS_)	ASAT volume estimated from partial ASAT area on *single slice* within rectangular box bound by either centers of femoral heads (*A*_p-FH_) or ASIS (*A*_p-ASIS_)
	*V*^~^_ASAT_(*A*_p-FH_)	
Partial volume	*V*^~^_ASAT_(*V*_p-ASIS_)	ASAT volume estimated from partial ASAT areas of *all slices* within rectangular box bound by either centers of femoral heads (*V*_p-FH_) or anterior superior iliac spines (*V*_p-ASIS_)
	*V*^~^_ASAT_(*V*_p-FH_)	

*AWF* maximum paramedian distance between outer rectus sheath and skin surface, *HGF* maximum perpendicular distance between tangent to iliac plate and skin surface.

### Statistical analysis

A least-squares regression (fit) between surrogate measure *S* and total ASAT volume was performed according to the equation

1 $$V_{{{\text{ASAT}}}} = S \cdot m_{{\text{s}}} + b_{{\text{s}}}$$

with resulting fit parameters slope (*m*_s_) and intercept (*b*_s_). For a given surrogate measurement, total ASAT volume estimates, denoted with a tilde, for example, *V*^~^_ASAT_(*d*_ven_), *V*^~^_ASAT_(*A*_p-ASIS_) or *V*^~^_ASAT_(*V*_p-FH_), can then be computed with these conversion parameters. The predictive value of a surrogate measure was quantified with the coefficient of determination *R*^2^ and the standard deviation *s*_d%_ of the percent differences between estimated and fully measured volume (*V*^~^_ASAT_ – *V*_ASAT_)/*V*_ASAT_ × 100%. All statistical analyses were performed with SPSS version 24 (IBM, Armonk, NY).

## Results

**Figure 3:**
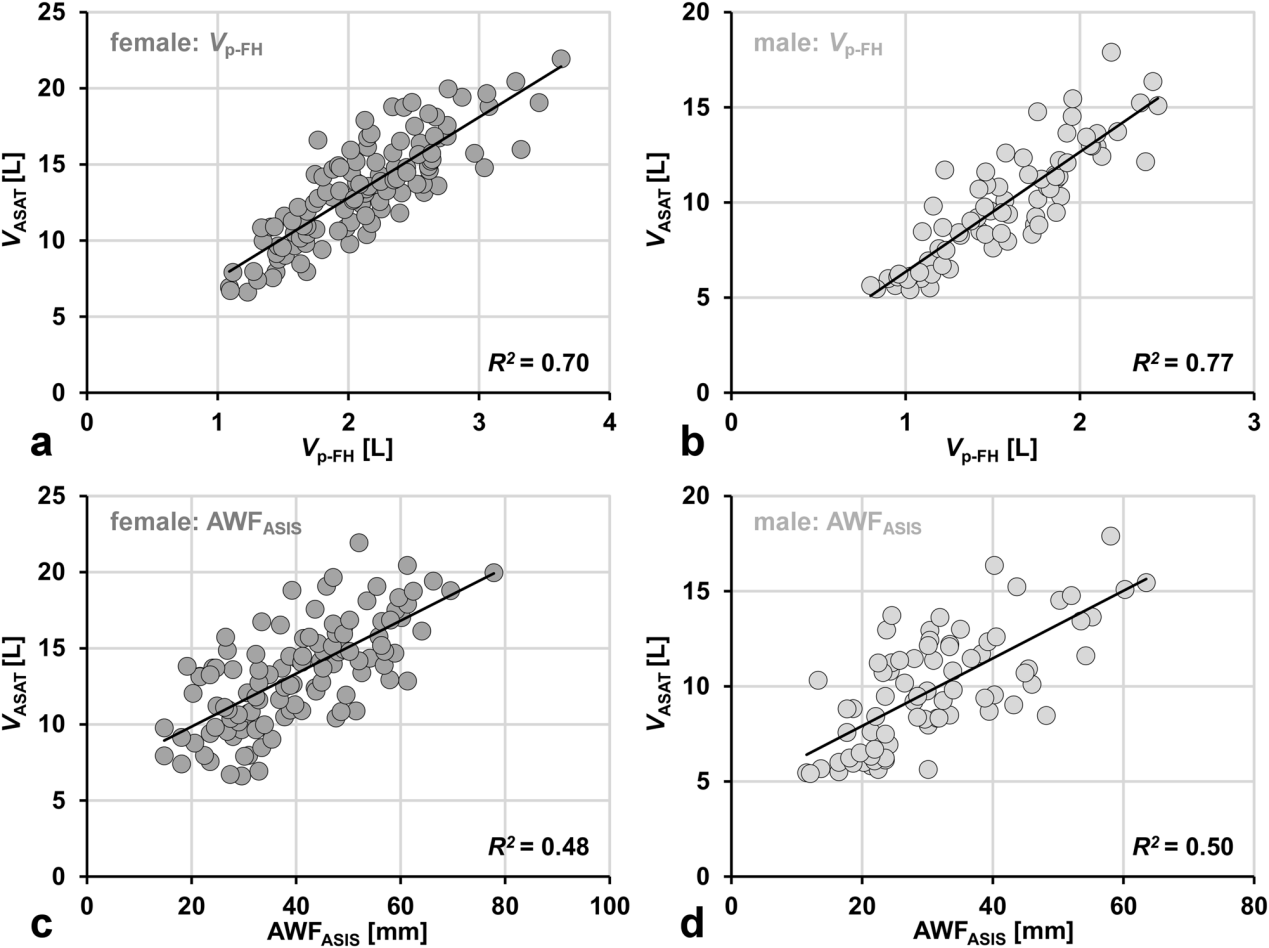
Gender-specific scatter plots and linear fits (solid lines) between reference ASAT volume (*V*_ASAT_) and two surrogate measures. (**a**, **b**) ASAT volume estimated from partial ASAT areas of *all* axial (10-mm thick) abdominal slices within rectangular box bound by centers of femoral heads (*V*_p-FH_). (**c**, **d**) simple length (thickness) of abdominal wall fat (AWF_ASIS_) on *single* slice at the level of the anterior superior iliac spine (ASIS). Plots are annotated with corresponding coefficient of determination *R*^2^.

Figure 3 shows gender-specific scatter plots and linear fits between fully measured ASAT volume and two selected surrogate measures: partial ASAT volume bound by femoral heads (*V*_p-FH_) and simple abdominal wall fat at the level of the anterior superior iliac spine (AWF_ASIS_). There is generally good to moderate agreement of the data with the linear fits. The coefficients of determination *R*^2^ are considered as good for *V*_p-FH_ and moderate for AWF_ASIS_. For both measures, the mere *R*^2^ values were slightly higher for males.

The resulting slope *m*_s_ and intercept *b*_s_ were used to compute estimated total ASAT volumes from the respective surrogate measures. Supplementary Fig. S1 shows the corresponding Bland–Altman plots between estimated and fully measured total ASAT volumes. Correspondingly, the standard deviation (and limits of agreement) are smaller for *V*_p-FH_ (vs. AWF_ASIS_) and also smaller for males (vs. females) for both surrogates.

**Figure 4:**
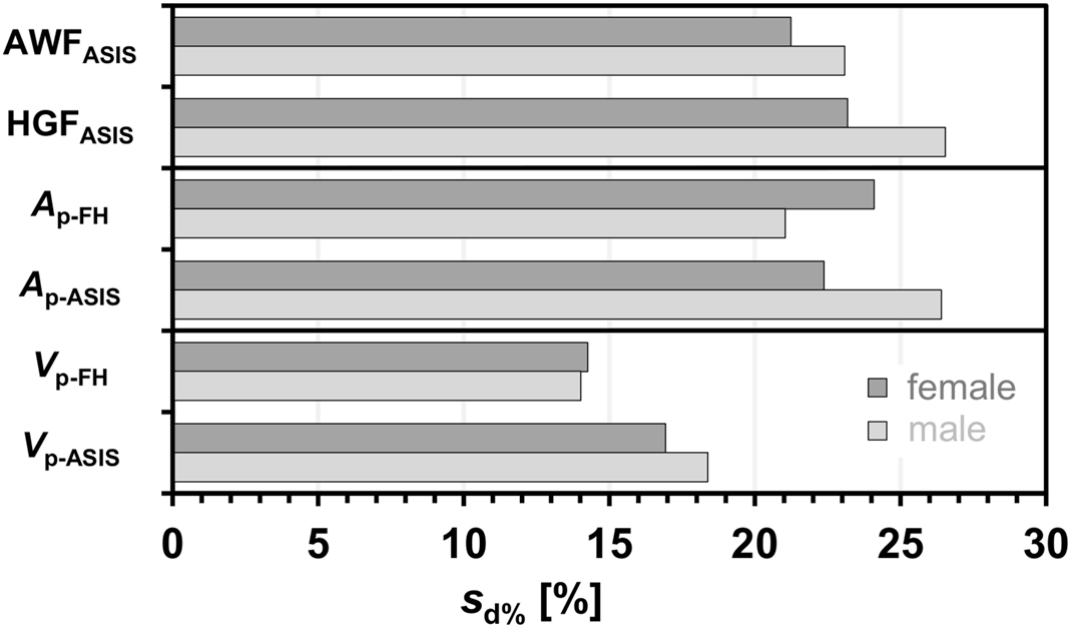
Standard deviations [%] *s*_d%_ of the mean differences between ASAT volume estimated from surrogate parameters (*V*^~^_ASAT_) and fully measured one (*V*_ASAT_) for both genders individually: simple lengths (AWF_ASIS_, HGF_ASIS_), partial areas (*A*_p-FH_, *A*_p-ASIS_) and partial volumes (*V*_p-FH_, *V*_p-ASIS_). ASIS anterior superior illiac spine, FH femoral heads.

Each difference was then divided by the respective reference ASAT volume to account for the different volume scales for females and males. The resulting standard deviations of the percent differences are plotted in Fig. 4 for all surrogate measures. At about 14%, *V*_p-FH_ represents the best surrogate followed by *V*_p-ASIS_ at around 17.5%. The *s*_d%_ values of partial areas and simple distances were all above 20% with the next best agreement for AWF_ASIS_. Gender differences were not systematic and on the order of a few percents except for *V*_p-FH_.

Table 2 provides a summary of all results and allows for a detailed inspection of both measures of agreement (*R*^2^ and *s*_d%_) and gender differences. *V*_p-FH_ happens to be the surrogate with both smallest *s*_d%_ and highest coefficient of determination as well as no substantial difference between females and males. By design, the reported *m*_s_ and *b*_s_ values represent the best fit to the respective data. In practice, however, they should only be used with the knowledge of the corresponding measures of agreement given here.

**Table 2:** Gender-specific measures of agreement and regression parameters between reference ASAT volume and surrogate ASAT measures.

Surrogate	Males (77)				Females (116)				Total (193)			
	*r*_p_ (*R*^2^)	*s*_d%_ [%]	*m*_S_	*b*_s_ [L]	*r*_p_ (*R*^2^)	*s*_d%_ [%]	*m*_s_	*b*_s_ [L]	*r*_p_ (*R*^2^)	*s*_d%_ [%]	*m*_s_	*b*_s_ [L]
AWF_ASIS_	0.69 (0.48)	21.2	0.174 L/mm	6.36	0.71 (0.50)	23.1	0.178 L/mm	4.36	0.73 (0.54)	23.9	0.198 L/mm	4.74
HGF_ASIS_	0.55 (0.30)	23.2	0.127 L/mm	4.58	0.62 (0.39)	26.5	0.162 L/mm	2.86	0.69 (0.47)	24.6	0.137 L/mm	3.95
*A*_p-FH_	0.50 (0.25)	24.1	0.127 L/cm^2^	6.05	0.73 (0.54)	21.0	0.198 L/cm^2^	2.05	0.69 (0.47)	23.4	0.164 L/cm^2^	3.70
*A*_p-ASIS_	0.60 (0.36)	22.4	0.097 L/cm^2^	5.13	0.57 (0.32)	26.4	0.090 L/cm^2^	4.49	0.69 (0.47)	24.5	0.107 L/cm^2^	3.97
*V*_p-FH_	0.84 (0.70)	14.3	5.303	2.19	0.88 (0.77)	14.0	6.297	0.06	0.88 (0.78)	14.4	5.774	1.07
*V*_p-ASIS_	0.79 (0.63)	16.9	3.430	2.98	0.77 (0.60)	18.4	3.850	1.04	0.83 (0.69)	18.1	3.794	1.59

*r*_p_, Pearson correlation coefficient; *R*^2^, coefficient of determination; *s*_d%_, standard deviation of the percent differences; *m*_s_ and *b*_s_, slope and intercept of linear regression.

Supplementary Table S3 provides a summary of literature results and recommendations for *V*_ASAT_ quantification from five MRI and CT studies in comparison with the present work. None of them, however, has explored the use of partial areas and volumes and proper measures of agreement (like *R*^2^) are only reported for one rather specific study.

## Discussion

The current guidelines for assessing body fat distribution in obesity are mainly based on measurements of BMI and waist circumference. These quantitative measures continue to provide researchers and clinicians with some basic information but cannot be used to distinguish a healthy metabolic phenotype from one with an elevated cardiometabolic risk, in particular at an early stage.

Blüher et al., for example, have described metabolically healthy obesity (MHO) as a subentity in which excessive body fat accumulation does not lead to adverse metabolic effects^32^. Such subjects are characterized by an increased expandability of SAT that may be accompanied by a lower fat deposition in visceral or ectopic regions. A personalized therapy therefore calls for a reliable method to quantify the individual abdominal fat compartments.

Fat content and distribution are increasingly considered as predictive biomarkers in longitudinal studies of obesity. Monitoring abdominal fat contents during different types of weight-loss interventions (behavior, diet, drug, surgery) is also likely to contribute to the understanding of obesity despite the complexity of the disease. Image segmentation, however, is usually time consuming and may not be appropriate for larger cohorts or clinical settings. A rapid yet reliable quantification method is highly desirable and various works have already investigated the power of selected single-slice data for the prediction of whole-abdominal fat volume.

In both a clinical drug trial over 24 weeks^27^ as well as a 6-month exercise intervention^29,33^, however, single-slice MRI fat estimates were found to be inappropriate to assess the changes in intraabdominal fat volume. For such trials, especially those involving weight loss, multi-slice MRI measurements seem to be inevitable at the moment^28–31^. Maurovich-Horvat et al. attributed this shortcoming to the inter-individual heterogeneity of the abdominal fat distribution and a different axial dimension of the abdomen. A planimetric assessment of the SAT/VAT ratio was found to be substantially different from a volumetric one^30^. Another study by Kanaley et al. also showed a poor prediction of total abdominal fat volume and related changes during weight loss^31^.

The present work provides a comprehensive quantitative evaluation of surrogate markers for the prediction of total ASAT volume in overweight and obese adults. Unlike previous studies in the literature, it specifically addresses the common challenge of image truncations which primarily occur in ventrolateral areas-given that patients are imaged in supine position. The measures of interest were therefore located dorsally except for abdominal wall fat thickness which was ventral but midsagittal.

The best correlation was observed for partial volume *V*_p-FH_ (Pearson's *r*_p_ = 0.88; *R*^2^ = 0.78) followed by *V*_p-ASIS_ (*r*_p_ = 0.83; *R*^2^ = 0.69) which is likely explained by the fact that volumetric measures encode more information about fat distributions than planimetric ones. Simpler measures like AWF_ASIS_, HGF_ASIS_, *A*_p-FH_ and *A*_p-SIAS_ with *s*_d%_ values over 20% are regarded as less useful. It is worth noting, that simple lengths at the ASIS level—*r*_p_/*R*^2^ (AWF) = 0.73/0.54 and *r*_p_/*R*^2^ (HGF) = 0.69/0.47—agreed better than any partial area (FH or ASIS). These lengths can be determined without much effort making them favorable for routine application. There is still a considerable improvement when using the best volumetric measure *V*_p-FH_ (from *r*_p_ = 0.73 to 0.88). Pausch et al., House et al., Li et al. and Raman et al. have already found ventral and dorsolateral lengths^27,34–36^ to be convenient and reproducible but did not correlate or validate them against a reference.

Total ASAT volume is a likely candidate as a tissue marker for metabolic, epidemiological or pharmaceutical studies. The present study is among the first ones to quantify the reliability of various surrogate measures for *V*_ASAT_. A look at five related studies (Table S3) shows that the focus until now has been on practical rather than methodological aspects. Most importantly, none of them have considered partial areas (or volumes) indicating that truncated datasets had been absent or excluded. A detailed comparison is therefore difficult.

The gender-specific conversion equations (12 in total) allow any researcher to compute ASAT volumes in a standardized manner, also retrospectively. The corresponding measures of agreement in Table 2 also indicate which combinations (gender and measure) tend to be more reliable (for example, seven cases with *r*_p_ around 0.7 or higher). Equations ignoring the gender are provided for comparison. For practical considerations, parameter *s*_d%_ appears to be more instructive than *R*^2^. All equations were derived with 10-mm thick slices, similar to other studies (Table S3).

Slice thickness will generally affect the tissue contours in the images (partial volume averaging) and consequently the exact equation. The overall effect for ASAT, however, should be moderate because the contours generally vary rather smoothly between transverse sections. Therefore, the provided equations should also hold approximately for slice thicknesses other than 10 mm which might be useful for other study designs.

This study is generally limited by its retrospective, single-center and mono-ethnic design. The results for men need to be interpreted with more care due to the lower number of participants. Manual segmentation is increasingly accepted as a reference standard despite the known variability between readers. One selection bias comes up because datasets were taken from existing subpopulations. An information bias towards lower degrees of obesity or overweight is inevitably introduced by requiring datasets with fully contained ASAT for validation.

In conclusion, partial subcutaneous fat volumes bound by the femoral heads seem to provide an acceptable workaround for whole-abdominal ASAT volumetry in subjects with truncated datasets frequently encountered for higher degree of obesity. The abdominal wall fat thickness at the ASIS level may be used with some reservation in cases where the amount of time for analysis is of importance.

## Supplementary information

Supplementary Information.

Supplementary Figure S1.

Supplementary Table S1.

Supplementary Table S2.

Supplementary Table S3.
