# Supplementary material for: Abdominal subcutaneous fat quantification in obese patients from limited field-of-view MRI data
Source: Sci Rep. 2020 Nov 4;10:19039. doi: 10.1038/s41598-020-75985-8 (PMC7642377; doi:10.1038/s41598-020-75985-8)
Supplement: Supplementary file 1 — Supplementary Information. [file 41598_2020_75985_MOESM1_ESM.docx]

Supplementary information

**Abdominal subcutaneous fat quantification in obese patients from limited field-of-view MRI data**

Sophia Michel ^1 #^, Nicolas Linder ^1 2 #^,Tobias Eggebrecht ^1 2^, Alexander Schaudinn ^2^, Matthias Blüher ^1 3^, Arne Dietrich ^1 4^, Timm Denecke ^2^, Harald Busse ^2^

^1^ Integrated Research and Treatment Center (IFB) Adiposity Diseases, Leipzig University Medical Center, Leipzig, Germany

^2^ Department of Diagnostic and Interventional Radiology, Leipzig University Hospital, Leipzig, Germany

^3^ Department of Internal Medicine, Neurology and Dermatology, Division of Endocrinology and Nephrology, Leipzig University Hospital, Leipzig, Germany

^4^ Department of Visceral, Transplantation, Thoracic and Vascular Surgery, Division of Bariatric Surgery, Leipzig University Hospital, Leipzig, Germany

^#^ Both authors have contributed equally to this work.
