# Supplementary figures and images for: Abdominal subcutaneous fat quantification in obese patients from limited field-of-view MRI data
Source: Sci Rep. 2020 Nov 4;10:19039. doi: 10.1038/s41598-020-75985-8 (PMC7642377; doi:10.1038/s41598-020-75985-8)

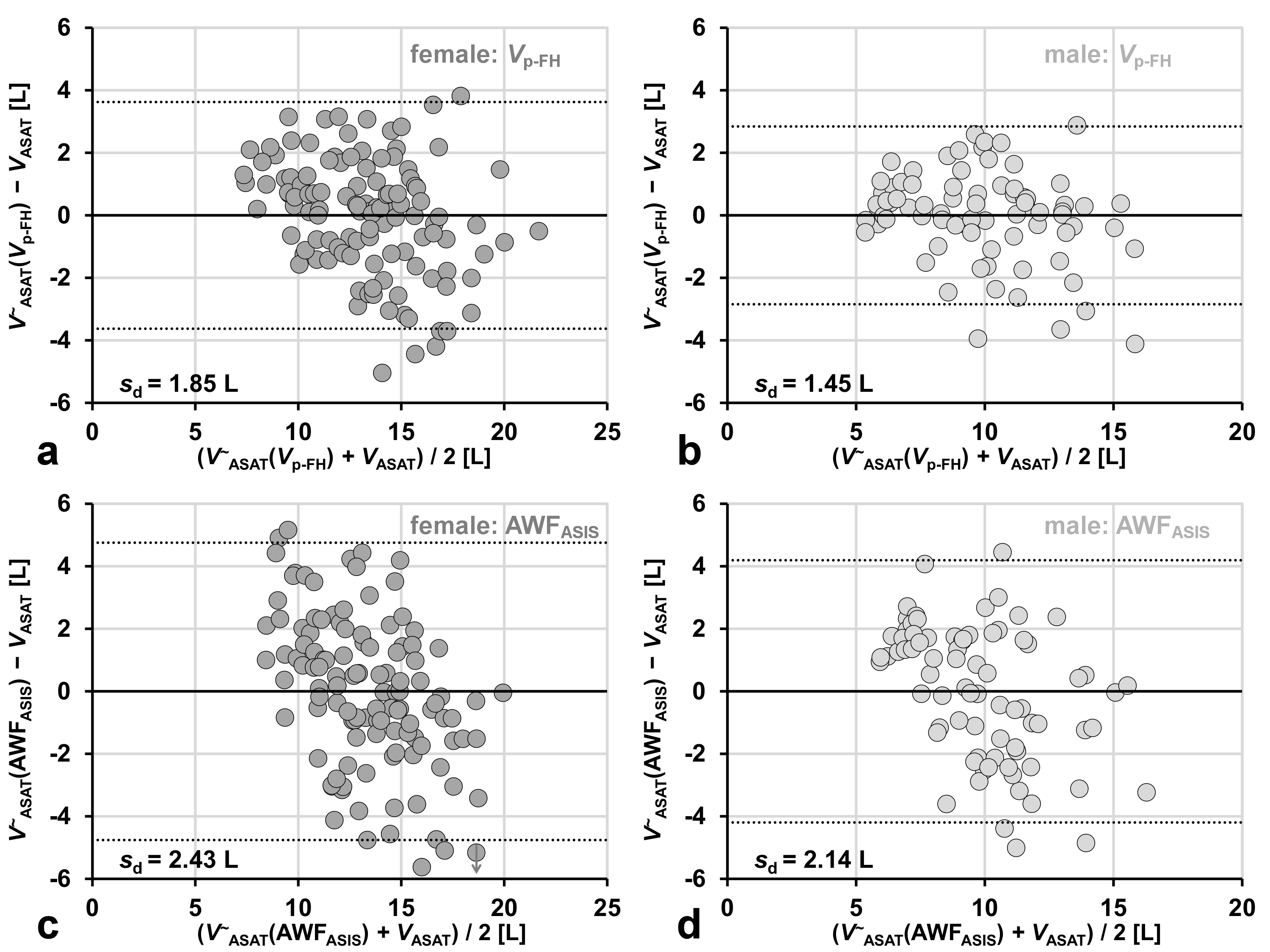

Supplement: Supplementary file 2 — Supplementary Figure S1. [file 41598_2020_75985_MOESM2_ESM.tif]
